# Accounting for Precision Uncertainty of Toxicity Testing: Methods to Define Borderline Ranges and Implications for Hazard Assessment of Chemicals

**DOI:** 10.1111/risa.13648

**Published:** 2020-12-09

**Authors:** Silke Gabbert, Miriam Mathea, Susanne N. Kolle, Robert Landsiedel

**Affiliations:** ^1^ Department of Social Sciences Wageningen University & Research Hollandseweg 1 Wageningen 6700 EW The Netherlands; ^2^ BASF SE Ludwigshafen am Rhein Germany

**Keywords:** Borderline range, classification threshold, decision‐making, OECD test guideline, precision uncertainty, prediction, toxicity testing

## Abstract

For hazard classifications of chemicals, continuous data from animal‐ or nonanimal testing methods are often dichotomized into binary positive/negative outcomes by defining classification thresholds (CT). Experimental data are, however, subject to biological and technical variability. Each test method's precision is limited resulting in uncertainty of the positive/negative outcome if the experimental result is close to the CT. Borderline ranges (BR) around the CT were suggested, which represent ranges in which the study result is ambiguous, that is, positive or negative results are equally likely. The BR reflects a method's precision uncertainty. This article explores and compares different approaches to quantify the BR. Besides using the pooled standard deviation, we determine the BR by means of the median absolute deviation (MAD), with a sequential combination of both methods, and by using nonparametric bootstrapping. Furthermore, we quantify the BR for different hazardous effects, including nonanimal tests for skin corrosion, eye irritation, skin irritation, and skin sensitization as well as for an animal test on skin sensitization (the local lymph node assay, LLNA). Additionally, for one method (direct peptide reactivity assay) the BR was determined experimentally and compared to calculated BRs. Our results demonstrate that (i) the precision of the methods is determining the size of their BRs, (ii) there is no “perfect” method to derive a BR, alas, (iii) a consensus on BR is needed to account for the limited precision of testing methods.

## 1 INTRODUCTION: PRECISION UNCERTAINTY OF TOXICITY TESTING METHODS

For hazard classifications of chemicals, continuous data obtained from animal tests or from nonanimal testing methods are dichotomized into binary “positive”/”negative” conclusion by applying thresholds (or cut‐offs). Experimental data obtained from a test method can be subject to different types of uncertainty, in particular interassay variability (i.e., the variability of test results between different laboratories; see, for example, Agnese, Risso, & De Flora, 1984; Hothorn, 2002, 2003; Richter, Garner, & Würbel, 2009), and intra‐assay variability (i.e., the ability of a test to reproduce its predictions). Intra‐assay variation can manifest in different ways. First, a method may not be able to reproduce results obtained from a reference test due to, for example, uncertainty in the reference data, of the hazard classification threshold assumed, or due to limitations of the prediction model. Second, tests may show limited reproducibility (or reliability) of test results in repeated applications. Assessing a test's reproducibility is, therefore, a core aspect of formal validation (Worth & Balls, 2002; Luechtefeld, Marsch, Rowlands, & Hartung, 2018). Though both problems have been acknowledged for a long time (Bruner, Carr, Chamberlain, & Curren, 1996; Hoffmann & Hartung, 2005; Pham et al., 2019), systematic research on how to overcome the latter type of uncertainty has started up just recently.

Specifically, Luechtefeld, et al. (2018) analyzed the reproducibility of OECD animal test guideline tests for several endpoints (acute oral and dermal toxicity, skin irritation, eye irritation, skin sensitization, and mutagenicity) using machine learning methods. Dumont, Barroso, Matys, Worth, and Casati (2016); Hoffmann (2015); Kolle et al. (2013), and Dimitrov et al. (2016) analyzed the reproducibility of the local lymph node assay (LLNA, OECD TG 429), being the reference test for assessing skin sensitization hazard. Kolle et al. (2013) showed that for those substances for which the estimated concentration (EC) leads to a simulation index (SI) value which was relatively close to the threshold for hazard classification (i.e., SI = 3; Kolle et al., 2013), repeated testing resulted in positive and negative classifications of their skin sensitization potential. Kolle et al. (2013) defined a range around the classification threshold of the LLNA, within which discordant outcomes can be expected, by determining coefficients of variation based on individual animal data. This range has been called “borderline range” (BR) (Kolle et al., 2013) or “grey zone” (Dimitrov et al., 2016). The percentage of discordant results was found to be greater when reference substances were tested with different vehicles (Hoffmann, 2015).

For skin sensitization hazard assessment, Leontaridou et al. (2017) compared the BR quantified for the LLNA with outcomes obtained from analyses of selected non‐animal tests, that is, the direct peptide reactivity assay (DPRA) OECD TG 442C, the antioxidant response element—nuclear factor erythroid 2 (ARE‐Nrf2) luciferase test methods covered by LuSens OECD TG 442D, the human cell line activation test (h‐CLAT) (OECD TG 442E), and a combination of the DPRA, LuSens, and the h‐CLAT into the “2‐out‐of‐3” defined approach (Bauch et al., 2012; OECD, 2016a, 2016; Urbisch et al., 2015 see also Sauer et al., 2016). Following the approach suggested in Kolle et al. (2013), the BR was defined to be the range around the classification threshold of the non‐animal testing method plus/minus one pooled standard deviation (p*SD*) of a testing method's results. Applying this method revealed percentages of substances considered borderline between 6% and 28% for the individual non‐animal methods, and 10 % for the “2‐out‐of‐3” defined approach, respectively. These results underline that a toxicity tests’ precision uncertainty due to intra‐assay variability can be nonmarginal. Furthermore, precision uncertainty needs to be taken into account when evaluating the reproducibility and predictive performance of the animal tests and of non‐animal methods, which are usually evaluated relative to the performance of the animal test (Paparella et al., 2013, 2017). Consequently, unraveling precision uncertainty can provide additional information that is relevant for evaluating a test's predictive performance, for example in a validation study and in OECD test guidelines.

So far, the BR has been explored for few testing methods only, that is, one reference test and selected non‐animal methods used for skin sensitization hazard identification. Thus, evidence on the existence and size of the BR is still limited. Furthermore, the size of the BR may depend on the method used for data analysis. The parametric approach used for determining the BR in the abovementioned studies implicitly assumes hazard classification results to be normally distributed. This may be a simplification and cannot be generalized for a broader class of reference tests and nonanimal methods.

The aim of this article is to go beyond existing research by evaluating and comparing different approaches to quantify the BR. This provides comprehensive insight into the patterns of precision uncertainty across toxicity tests and offers a pragmatic approach how to deal with precision uncertainty of testing methods. First, we quantify the BR for non‐animal tests for different endpoints, including skin corrosion, eye irritation, skin irritation, and skin sensitization. Second, the BR is quantified with both parametric and nonparametric methods. Besides using the p*SD*, we determine the BR by means of the median absolute deviation (MAD), with a sequential combination of both methods, and by using nonparametric bootstrapping. Finally, we apply the analysis on different sets of experimental data to analyze the effect of data set composition on the size of the BR.

## 2 STATISTICAL METHODS FOR QUANTIFYING THE BORDERLINE RANGE OF TESTING METHODS

A toxicological test is a controlled experiment that is designed in order to detect a certain hazardous effect of a chemical. Irrespective whether a testing method is an animal experiment, that is, using living organisms (*in vivo*, with vertebrates or invertebrates), or a non‐animal test using, for example, cell lines (*in vitro*) or computational methods (in silico), it generates continuous experimental data to which a prediction model using a classification threshold (CT) is applied in order to discriminate between chemicals that are classified as hazardous (i.e., to show a certain effect; “positive”) and those that are not (“negative”). The definition of such CT is therefore a core element of the statistical evaluation model of a test method. The present section introduces four statistical methods for determining the BR around the CT of a toxicity test, if the CT is exogenous (Leontaridou et al., 2017). Here we discuss the basic principles of each method and explain how the BR is quantified. Additional information on the computational approach can be found in the Appendix.

Following the explanation of the approaches to quantifying the BR, we provide an overview of the non‐animal toxicity tests that were used for generating experimental data on chemical hazards.

### 2.1 Pooled Standard Deviation

The p*SD* is a weighted average of the standard deviations of different data sets having different sizes. As explained in Leontaridou et al. (2017), the pooling is across substances and concentrations. Subtracting the pooled *SD* from the classification threshold, and adding it to the threshold, reveals the BR. However, since the *SD* is very sensitive to outliers and to nonnormality in the distribution of data, results of the *SD* are not very robust.

### 2.2 Pooled Median Absolute Deviation

The mean average deviation (MAD) offers another, more robust scale estimator. Based on the MAD method, the BR is computed analogously to the p*SD*, but from the pooled median absolute deviation of a test method's results (see Appendix).

In general, scale estimators can break down in two ways, that is, when they become arbitrarily large (explosion) or when they become close to zero (implosion). In the case of explosion this would lead to an arbitrarily wide borderline range, whereas implosion would cause the scale estimator to become arbitrarily narrow. Naturally, controlling for explosion as well as implosion is important. For *n* = 3 it is impossible to estimate the scale robustly, that means protection against implosion and explosion is not possible at the same time. For *n* ≥ 4, the scale can be determined robustly. In that case, the MAD has the maximal breakdown value and, hence, it is the recommended scale estimator (Rousseeuw & Verboven, 2002). Since in this context explosion is more critical, it is recommended to use the MAD. Similar to quantifying the BR using the p*SD* , the BR around the prediction model's classification threshold is given by subtracting and adding the MAD from/to the threshold.

### 2.3 Confidence Interval Approximation of the Pooled Median Absolute Deviation Using the Bootstrap Percentile Method

Like all bootstrap methods, the percentile bootstrap does not make assumptions about the underlying distributions from which our observations could have been sampled. The data themselves are used to estimate sampling distributions (Efron & Tibshirani, 1993; Mooney et al., 1993; Ng & Wilcox, 2011). The method uses resampling with replacement to generate an approximate sampling distribution of an estimate.

The percentile method is often used to provide an approximate 95% confidence interval for the population parameter. In this case the parameter is the pooled median absolute deviation. The percentile method is not as accurate as other bootstrap methods, but it is straightforward to calculate. Suppose 1,000 bootstrap replications are collected. After ranking from bottom to the top the 90% confidence interval (CI) is obtained by reading out the sample quantiles corresponding to the given probabilities (0.1; 0.9). In our analysis the upper limit of the 90% confidence interval is used. Hence, the BR is computed by subtracting and adding the 90% confidence interval to the threshold.

### 2.4 The 90% Percentile of All MADs

The methods discussed in the previous sections considered pooled values. Hence, the quantification of the BR is based on mean estimates over the entire set of experimental data. This implies that it is possible to characterize the uncertainty only if, on average, a new value (range) may overlay the classification threshold. However, if it is of interest whether repeated measurements of a new substance are likely to deliver discordant results, the borderline range from the former introduced methods might be too small. Instead of averaging experimental estimates the borderline range should be computed based on the 90% percentile of the distribution of MADs. This implies that the borderline range contains 90% of all computed MADs and is computed by deviating a 90% percentile of all MADs on both sides of the threshold. Details about the computation of the BR using the 90% percentile method are provided in the Appendix.

## 3 EMPIRICAL APPLICATION

### 3.1 Selection of Testing Methods for Determining the BR

The BR was quantified based on data sets of nine test methods, addressing different types of hazardous effects caused to the eyes and the skin of humans, following an acute exposure to a chemical (skin sensitization, skin corrosion, skin irritation, and eye irritation). Of the nine test methods considered, eight tests were non‐animal tests. Table I provides an overview of the corresponding OECD test guidelines (TG), including the addressed endpoint, the prediction model, and the prediction models’ classification threshold used for detecting an effect. The table also indicates whether or not a *BR* has been included in the OECD TG.

**Table I:** Overview of Test Methods Used for Determining the BR of Their Prediction Models

Method Name (abbreviation)	Hazardous Effect/Endpoint	Animal/Nonanimal Test	OECD Test Guideline (TG); Reference	BR in Test Guideline?	Prediction Model's Classification Threshold
Local lymph node assay (LLNA)	Skin sensitization	Animal test	429: OECD (2010)	No	Skin sensitizing potential if stimulation index (SI) of ^3^H‐thymidine incorporation ≥ 3 compared to the concurrent vehicle control group
Direct Peptide Reactivity Assay (DPRA)	Skin sensitization	Nonanimal (*in chemico*)	442C; OECD (2019a)	Mean Lys/Cys^b^ peptide depletion 3–10%; Cys^b^ depletion 9–17%	Positive if mean of Lys^b^ and Cys^b^ peptide depletion >6.38%, or Positive if Cys‐depletion >13.89%
LuSens assay	Skin sensitization	Nonanimal (*in vitro*)	442D; OECD (2018a)	No	Positive if statistically significant induction of the luciferase activity > 1.5
Human Cell Line Activation Test (h‐CLAT)	Skin sensitization	Nonanimal (*in vitro*)	442E: OECD (2018b)	No	Positive if cell surface markers CD54 and CD86 show an expression above 2.0‐fold and/or 1.5‐fold, respectively, after test substance treatment relative to concurrent vehicle controls and at relative cell viabilities of at least 50%
EpiDerm Skin Corrosion test (SCT)	Skin corrosion	Nonanimal (*in vitro*)	431; OECD (2019b)	No	Corrosive if relative cell viability < 50% after 3 minute exposure and/or < 15% after one hour exposure
Corrositex®	Skin corrosion	Nonanimal (*in vitro*)	435; OECD (2015a)	No	*High acid/alkaline reserve (categorization screen category 1)*: Corrosive optional category 1A if mean breakthrough time of 0–3 minutes. Corrosive optional category 1B if mean breakthrough time > 3 and < 60 minutes. Corrosive optional category 1C if mean breakthrough time > 60 and < 240 minutes. Noncorrosive if mean breakthrough time > 240 minutes. *Low acid/alkaline reserve (categorization screen category 1)*: Corrosive optional category 1A if mean breakthrough time of 0–3 minutes. Corrosive optional category 1B if mean breakthrough time > 3 and < 30 minutes. Corrosive optional category 1C if mean breakthrough time > 30 and < 60 minutes. Noncorrosive if mean breakthrough time > 60 minutes.
EpiDerm® Skin Irritation test (SIT)	Skin irritation	Nonanimal (*in vitro*)	439: OECD (2019c)	Yes (45–55%)	Irritant if relative cell viability is < 50%.
Bovine corneal opacity and permeability test (BCOP)	Eye irritation	Nonanimal (*in vitro*)	437; OECD (2017)	Yes^a^	Seriously eye damaging if in vitro irritation score (IVIS) > 55 No prediction can be made if 3 < IVIS ≤ 55 Nonirritant if IVIS ≤ 3
EpiOcular® Eye Irritation test (EIT)	Eye irritation	Nonanimal (*in vitro*)	492: OECD (2019d)	Yes (55–65%)	Nonirritant to the eye if relative cell viability is > 60%

^a^ No numerical BR is described in OECD TG 437, but it is described that a “testing run is considered borderline if the predictions from the three corneas were nonconcordant, such that (i) two of the three corneas gave discordant predictions from the mean of all three corneas, OR, (ii) one of the three corneas gave a discordant prediction from the mean of all three corneas, and the discordant result was >10 IVIS units from the cut‐off threshold of 55. If the repeat testing run corroborates the prediction of the initial testing run (based upon the mean IVIS value), then a final decision can be taken without further testing. If the repeat testing run results in a nonconcordant prediction from the initial testing run (based upon the mean IVIS value), then a third and final testing run should be conducted to resolve equivocal predictions, and to classify the test chemical. It may be permissible to waive further testing for classification and labeling in the event any testing run results in a UN GHS Category 1 prediction,” cf. OECD (2017).

^b^ Lys/Cys: Lysine and cysteine.

The sizes of the data sets (all experimental data used in the current study was generated during routine testing in the GLP and ISO 17020 certified laboratories of BASF SE's experimental toxicology and ecology, Germany) and the cut‐off values for each method are given in table (Table II). For those evaluations where the natural logarithm was taken data points below and equal to zero were removed from the data sets.

### 3.2 Experimental Validation of Derived Borderline Ranges: The Example of the DPRA Test

The BR is the variability of a testing methods experimental result at the CT. We derived the BR by using the variability of over the full range of experimentally derivable results (e.g., 0–100% peptide depletion in the case of the DPRA). We then used calculation methods to approximate the variability at the CT. In contrast, repeated testing of a test substance which yields testing results at (or close by) the CT allows quantifying the actual BR. Comparing the derived BR with the actual variability of testing results at the CT is therefore a convenient method for validating methods. To provide an experimental validation of the BR we selected the DPRA (following OECD TG 442C) since it is the least resource‐intensive method in comparison to other testing methods in our sample. Besides the experimental validation of the DPRA borderline range, no additional experiments were conducted for the purpose of this study.

**Table II:** Borderline Ranges (BR) of Various *In Vitro* Methods and One *In Vivo* Method Quantified by Different Methods

					Borderline Range							
					Method 1	Method 2	Method 3	Method 4	Method 5	Method 6	Method 7	Method 8
Test Method	Test Result	Cut‐off	Tg Borderline Range	Data Set Size^a^	Pooled *SD*	log pooled *SD*	pooled MAD	log pooled MAD	MAD + bootstrap	log MAD + bootstrap	MAD + percentile	log MAD + percentile
Skin Corrosion/Irritation Tests												
EpiDerm SCT (OECD TG 431)	relative viability after 3 minutes exposure [%]	50	45–55	275	44–56	46 ‐ 55	46–54	47–53	45–55	47 ‐ 54	40–60	43–58
	relative viability after 60 min exposure [%]	15	10–20	281	8 –22	13–18	10–20	14–17	10–20	13–17	4–26	12–19
EpiDerm SIT (OECD TG 439)	relative viability after 60 minutes exposure [%]	50	45–55	718	38–62	35–71	45–55	45–55	45–55	45–56	39–61	40–62
Corrositex (OECD TG 435)	Corrositex Category 1^b^ break through time [min]	3	n/a	49	1.1–4.9	2.6–3.5	2.0–4.0	2.8–3.3	1.8–4.2	2.7–3.3	2.6–3.5	0.5–5.5
		60	n/a		58.1–61.9	51.2–70.3	59.0–61.0	55.2–65.2	58.8–61.2	54.0–66.7	51.6–69.7	57.5–62.5
		240	n/a		238.1–241.9	204.9–281.2	239.0–241.0	220.7–261.0	238.8–241.2	216.0–266.7	206.5–279.0	237.5–242.5
	Corrositex Category 2^b^ break through time [min]	3	n/a	5	‐6.2–12.2	2.7–3.3	0.7–5.3	2.9–3.2	2.0–4.0	2.9–3.1	‐2.08.0	2.7–3.3
		30	n/a		20.8–39.2	27.1–33.2	27.7–32.3	28.5–31.5	29.0–31.0	29.4–30.6	25.0–35.0	27.5–32.7
		60	n/a		50.8–69.2	54.3–66.3	57.7–62.3	57.1–63.1	59.0–61.0	58.8–61.2	55.0–65.0	55.0–65.0
Eye Irritation Tests												
EpiOcular EIT (OECD TG 492)	relative viability [%]	60	55– 65	907	55–65	50–72	56–64	54–67	56–64	53–68	51–69	47–77
BCOP (OECD TG 437)	IVIS	55	n/a^c^	814	46–64	29–125	51–59	38–80	50–60	35–71	46–64	22–136
	IVIS	3	n/a^c^		1–7	‐6–12	‐1–7	2–4	‐2–8	2–4	‐6–12	1–7
Skin Sensitization Tests												
DPRA (OECD TG 442C)	Mean peptide depletion [%]	6.38	3–10	385	4.13–8.63	4.33 ‐ 9.40	4.79–7.98	5.29–7.69	4.70–8.06	5.20–7.82	2.69–10.07	3.68–8.01
	Cysteine‐only depletion [%]	13.89	9–17		9.95– 17.83	9.82–19.64	11.15–16.63	11.62–16.61	11.00–16.78	11.47–16.82	7.99–19.79	8.20–23.52
LuSens (OECD TG 442D)	Luciferase fold induction	1.5	n/a	2,514	1.2– 1.8	1.3–1.7	1.4–1.6	1.4–1.6	1.4 ‐ 1.6	1.4–1.6	1.3–1.7	1.3–1.8
h‐CLAT (OECD TG 442E)	CD54 induction	200	n/a	2,379	‐1–401	158–254	125–275	170–235	121–279	170–236	51–349	142–283
	CD86 induction	150	n/a	2,415	127– 173	125–180	135–165	132–170	135–165	132–171	117–183	115–196
LLNA (OECD TG 429)	Thymidine incorporation	3	n/a	277	0.9–5.1	2.0–4.6	2.1–3.9	2.1–4.2	2.0–4.0	2.1–4.3	2.3–3.7	2.0–4.5
	Lymph node cell counts	1.5	n/a		1.2–1.8	1.2–1.9	1.3–1.7	1.3–1.8	1.3–1.7	1.3–1.8	0.8–2.2	1.0–2.2
	Lymph node weight	1.5	n/a		1.3–1.7	1.3–1.7	1.4–1.7	1.3–1.7	1.4–1.7	1.3–1.7	0.8–2.2	1.0–2.2
	Ear weight	1.25	n/a		1.19–1.31	1.18–1.32	1.21–1.30	1.20–1.30	1.20–1.30	1.19–1.31	0.59–1.91	0.84–1.87

^a^ All experimental data used in the current study was generated during routine testing in the GLP and ISO 17020 certified laboratories of BASF SE's Experimental Toxicology and Ecology, Germany.

^b^ The Corrositex Category is determined in the categorization screen. Depending on the outcome one or the other prediction model is used for the final prediction.

^c^ No numerical *BR* is described in OECD TG 437, but it is described that a “testing run is considered borderline if the predictions from the three corneas were nonconcordant, such that (i) two of the three corneas gave discordant predictions from the mean of all three corneas, OR, (ii) one of the three corneas gave a discordant prediction from the mean of all three corneas, AND the discordant result was >10 IVIS units from the cut‐off threshold of 55. If the repeat testing run corroborates the prediction of the initial testing run (based upon the mean IVIS value), then a final decision can be taken without further testing. If the repeat testing run results in a nonconcordant prediction from the initial testing run (based upon the mean IVIS value), then a third and final testing run should be conducted to resolve equivocal predictions, and to classify the test chemical. It may be permissible to waive further testing for classification and labeling in the event any testing run results in a UN GHS Category 1 prediction”, cf. OECD (2017).

Briefly, in the DPRA the reactivity of a test substance toward synthetic cysteine and/or lysine‐containing peptides is evaluated. For this purpose, the test substance is incubated with synthetic peptides for 24 hours at approximately 25 °C and the remaining nondepleted peptide concentration is determined thereafter by high performance liquid chromatography with gradient elution and UV‐detection at 220 nm. The peptide depletion of test‐substance incubated samples is compared to the peptide depletion of the negative control samples and expressed as relative peptide depletion.

Specifically, ethylene glycol dimethacrylate (EGDMA) was used as a “typical” test substance (poorly water‐soluble and a moderate or weak skin sensitizer (CLP Cat. 1B, LLNA EC3 28%). The concentrations of EGDMA (8 and 10 mM stock concentrations) were chosen to yield a test result being at or close to the CT, that is, 6.38% mean peptide depletion (the threshold of the DPRA prediction model). In total, nine independent runs per concentration with three replicates per run were performed (a total of two times 27 testing results or 54 data points for mean peptide depletion close to the threshold of 6.38%). The range of these 2 × 27 testing results defines the actual BR of this testing method (with this substance, in this laboratory) and can be compared to the BR derived by statistical methods using all the testing results.

## 4 RESULTS

### 4.1 Quantification of the BR Using Statistical Methods

**Fig 1:**
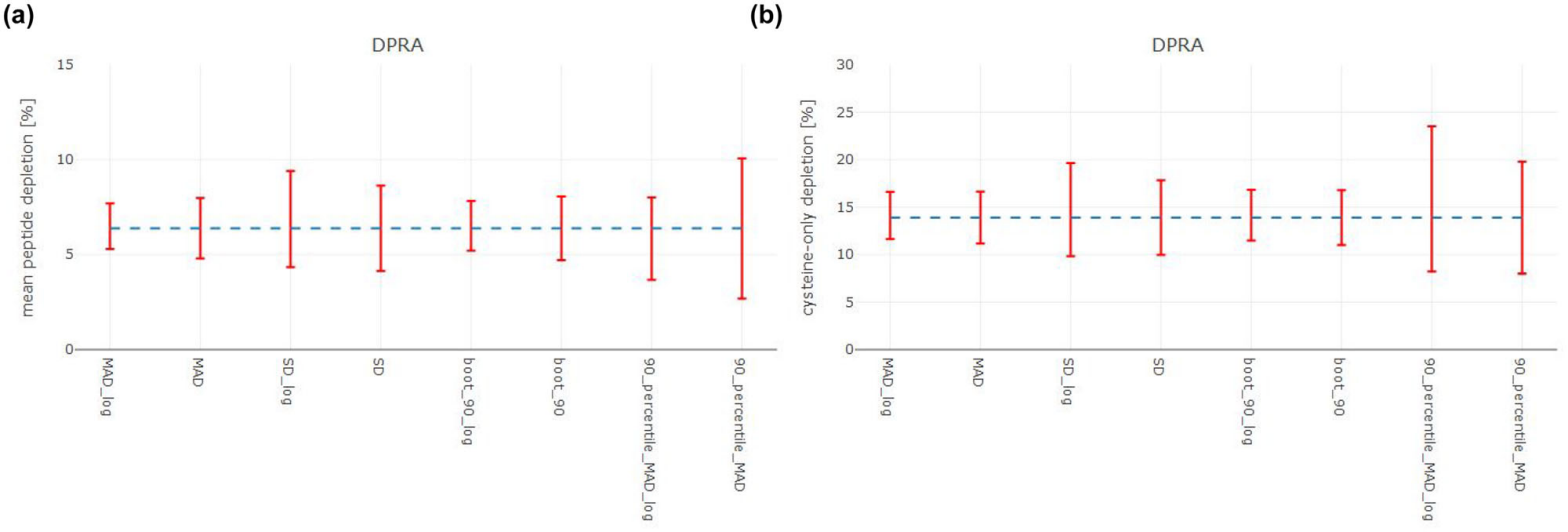
DPRA BRs for mean peptide depletion (panel A) and Cys‐only depletion (panel B) based on log transformed (indicated by “_log”) and nontransformed data of the mean absolute deviation (MAD), pooled standard deviation (SD), the bootstrapped method and the 90% percentile of all MADs. The interrupted line marks the cut‐offs as described in OECD TG 442C (i.e., 6.38% mean peptide depletion and 13.89% Cys‐only depletion) and the red lines indicate the upper and lower boundaries of the borderline range for the individual calculation methods. All experimental data used in the current study was generated during routine testing in the GLP and ISO 17020 certified laboratories of BASF SE's Experimental Toxicology and Ecology, Germany.

**Fig 2:**
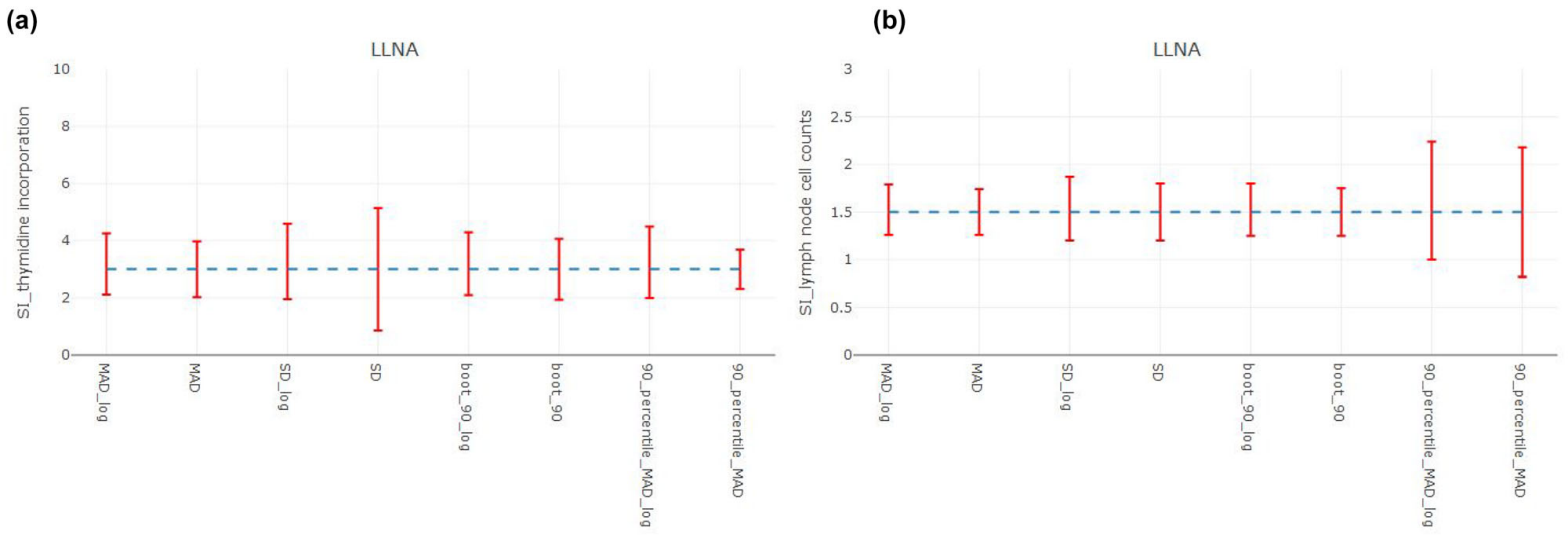
LLNA BRs for based on data of the stimulation index for thymidine incorporation (panel A) and the stimulation index for lymph node cell counts (panel B) based on log transformed (indicated by “_log”) and nontransformed data of the mean absolute deviation (MAD), pooled standard deviation (*SD*), the bootstrapped method and the 90% percentile of all MADs. The interrupted line marks the cut‐offs as described in OECD TG 429 (i.e., an SI of 3 for thymidine incorporation) and in Basketter et al. (2012). The red lines indicate the upper and lower boundaries of the borderline range for the individual calculation methods. All experimental data used in the current study was generated during routine testing in the GLP and ISO 17020 certified laboratories of BASF SE's Experimental Toxicology and Ecology, Germany.

**Fig 3:**
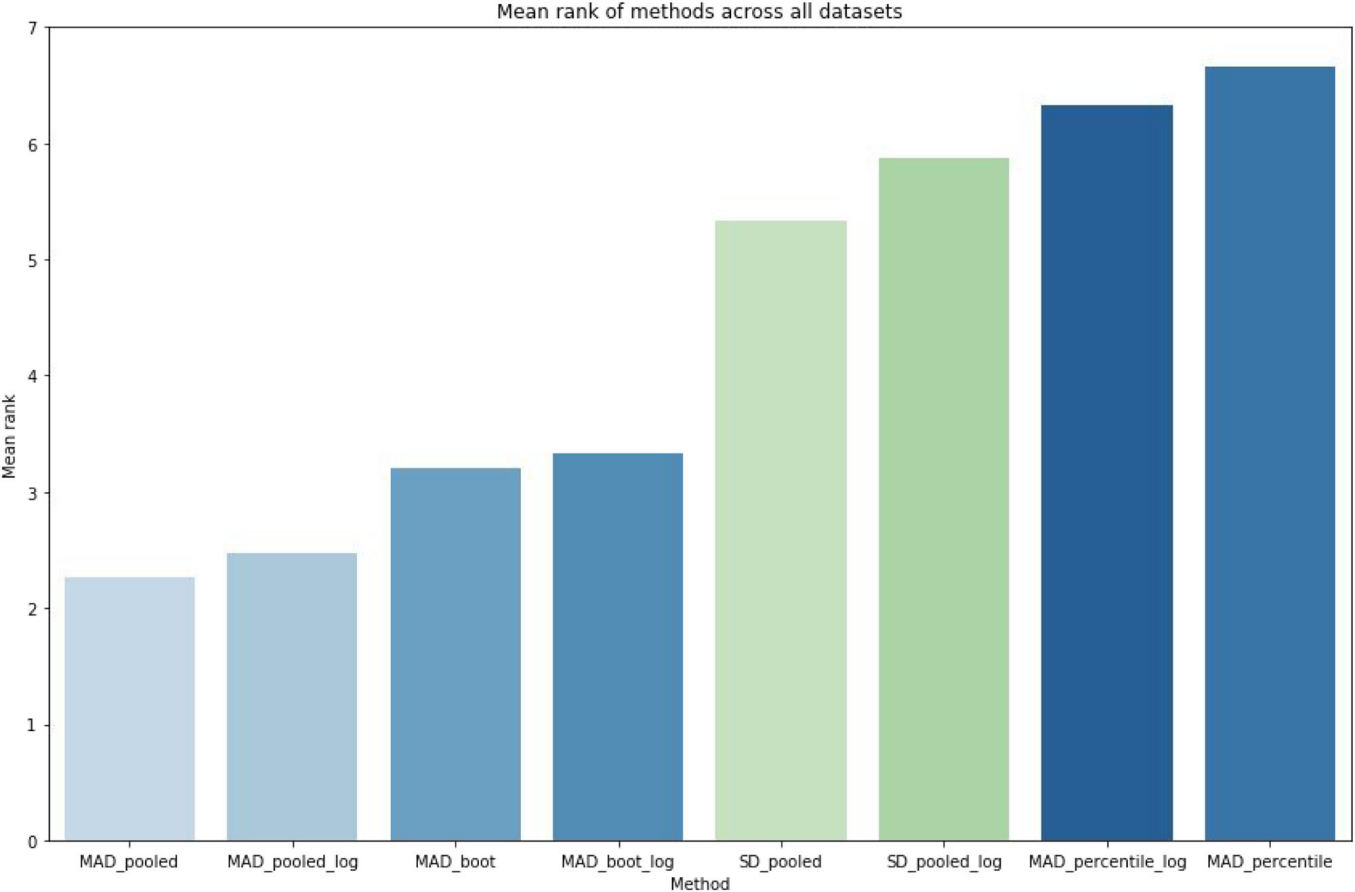
Mean rank of methods across all data sets based on log transformed (indicated by “_log”) and nontransformed data of the mean absolute deviation (MAD), pooled standard deviation (SD), the bootstrapped method and the 90% percentile of all MADs.

Fig. 1 shows the results of quantifying the BR with different methods using the DPRA data set. On the left‐hand side (A) the DPRA BRs for the mean peptide depletion are plotted. The right‐hand side (B) shows the BRs for the Cys‐only depletion, respectively. Fig. 2 visualizes the results obtained from using the LLNA data set. The left‐hand side (panel A) shows the LLNA BRs for the stimulation index for thymidine incorporation, the right‐hand side (panel B) the borderline ranges for the stimulation index for lymph node cell counts. The BRs are computed based on log transformed and nontransformed data of the mean absolute deviation (MAD), the pooled standard deviation (SD), the bootstrapped method and the 90% percentile of all MADs. The interrupted line in Fig. 1 marks the cut‐offs as described in OECD TG 442C (i.e., 6.38% mean peptide depletion and 13.89% Cys‐only depletion). The interrupted line in Fig. 2 highlights the cut‐offs as described in OECD TG 429 (i.e., an SI of 3 for thymidine incorporation) and Basketter et al., 2012 (i.e., and SI of 1.5 for the lymph node cell counts). The red error bars indicate the upper and lower boundaries of the borderline range for the individual calculation methods. The smallest borderline ranges for the DPRA and LLNA data set are computed based on the pooled MAD or bootstrapped MAD method and the pooled SD. The 90% percentile method, to the contrary, leads to wider borderline ranges. The use of log‐transformed data does not influence the ranking. These results are also representative for the other data sets and are shown by Fig. 3 (details of all combinations of methods and data sets can be found in the summary table.)

Fig. 3 illustrates the mean ranks of all methods across all data sets. In detail the method with the smallest BR gets rank 1 and so on, if a method's rank is 3* rank 3, 1* rank 2, 3* rank 1 and we consider seven data sets, then the mean rank would be 2. The two methods with the smallest BR are the pooled MAD and the variant including the bootstrap. In contrast, the method that uses the 90% percentile provides the greatest intervals. This could be expected based on the calculation, because no aggregated MADs are used to define the BR. The BR of the p*SD* is greater than the one based on the pooled MAD, because the former is more sensible to outliers than the pooled MAD. In general, the log transformation of the data decreases the size of the BR.

### 4.2 Experimental Validation of the Statistically Derived Borderline Ranges for the DPRA Test

The results of the experimental validation of the statistically derived borderline range for the DPRA are summarized in Table III. The experimental BR is smaller than any of the BRs derived by statistical methods. This would imply an overestimation of the BR by all calculation methods based on experimental data (Table II). Obviously, the DPRA with EGDMA concentrations yielding results close to the CT is more precise than with several other test substances close to and further away from the cut‐off. Hence, lower precision (and hence larger BR) arises from (i) testing results being farther away from the CT and/or (ii) testing different substances (instead of the same test substances). It should be noted, that the experimental BR indeed addresses the uncertainty of testing one test substance at the CT.

**Table III:** Experimental Borderline Range (BR) of DPRA Close to the Cut‐Offs at 6.38% and 13.89%, Respectively

Test Method	Test Substance Stock Concentration	Size of Data Set	Mean Test Result		Result Range [mean +*SD*, mean – *SD*]	Result Range linearly transformed to the cut‐offs at 6.38% and 13.89%, respectively[mean + *SD*, mean – *SD*]
DPRA (OECD TG 442C)	8 µM EGDMA	27	Mean peptide depletion 9%)	6.85	5.92–7.78	5.45–7.31
		27	Cysteine‐only depletion (%)	12.15	10.15–14.16	11.89–15.90
DPRA (OECD TG 442C)	10 µM EGDMA	27	Mean peptide depletion (%)	8.27	7.26–9.28	5.37–7.39
		27	Cysteine‐only depletion (%)	14.15	12.03–16.01	11.77–15.75

Calculation method 4 (which is based on log pooled MAD) gives, however, the closest approximation of the experimental data. Obviously, this is only valid for the DPRA and based on a limited experimental data set.

## 5 CONCLUSIONS AND IMPLICATIONS FOR HAZARD ASSESSMENT OF CHEMICALS AND THE VALIDATION OF NEW TESTING METHODS

For hazard identification ordinal and continuous read‐outs from testing methods are transformed into a dichotomous result (either “positive” or “negative”) by defining a cut‐off or classification threshold. Test substances with read‐outs close to this threshold could be assessed as positive or negative upon retesting due to technical and biological variability; thus, the result is ambiguous. Hence, the precision of toxicity testing methods in this range is limited. This precision uncertainty is often neglected in reporting and assessing the results. The BR offers a simple and pragmatic way to consider this uncertainty, and therefore lays the methodological grounds for taking precision uncertainty into account. Understanding precision uncertainty of chemical testing methods, therefore, supports a more transparent evaluation of the predictive accuracy of testing methods. It is well acknowledged that there are also other types of uncertainties, for instance uncertainties due to limitations in the experimental design of a test method, chemical‐specific applicability constraints, or bias in the specification of the classification threshold. The focus of the present study is, however, on the uncertainty around the classification threshold of regulatory accepted test methods. Since these are typically conducted in a GLP‐environment and/or following the OECD guidance document on good *in vitro* method practices (GIVIMP, OECD, 2018c), the abovementioned uncertainties can be assumed to be limited.

In this study the BR around the CT of a toxicity testing method's prediction model is quantified using different statistical methods. In addition, experimental data were used to validate the computed results and to discuss which method may fit best. Contrary to the current approach of defining a CT leading to binary classifications (toxic/nontoxic, hazardous/nonhazardous), a BR defines a range within which classification of experimental test results is inconclusive (hence resulting in three possible outcomes: positive, negative, and inconclusive). Thus, a BR is setting two new thresholds (between positive and inconclusive, and negative and inconclusive). Though this is a simplification compared to a fully probabilistic analysis (for which the toxicological and regulatory community may not be ready yet), it offers a simple and pragmatic way to address uncertainty of test results. Obviously, uncertainty is the smaller the larger the distance of an experimental result from the CT.

In this study data from one laboratory conducting the assays for routine purposes were used. Generally, we propose to have BRs to be statistically defined in the test guidelines. As a ring trial is always conducted before a test guideline is adopted, the BR could and should be determined from a multilab (reproducibility) ring trial.

Based on our analysis, we therefore propose that descriptions of testing methods, especially test guidelines, should acknowledge the consequences of testing methods’ limited precision. This includes

1. defining and reporting BR around the threshold of the prediction model which dichotomizes the read‐out, and
2. documenting three kinds of results: “positive,” “negative,” or “inconclusive”/“ambiguous” (within the BR).

Less precise testing methods will have larger BR than more precise methods and hence more test substances will yield inconclusive results. This incentivizes developing more precise methods (or expanding efforts to understand imprecisions of existing methods, see, for example, Alépée et al. (2005); Hoffmann et al. (2010); Spielmann et al. (2007). At the same time, it constitutes a need for supplementary testing methods in order to reach conclusions on results which are inconclusive by just one method.

It is common practice to characterize and assess a new test method's predictive accuracy by calculating the sensitivity, specificity, and concordance with data obtained by a reference method (the so‐called “gold standard” which is often the animal method). So far, however, uncertainties of these metrics have been largely ignored. This may cause bias in conclusions on an individual testing method's predictive accuracy. Including the BR of the new and the reference method may change our understanding of the relative performance of nonanimal testing methods in comparison to the reference data (Leontaridou, Gabbert, & Landsiedel, 2019). In fact, the BR should be considered when estimating the concordance of experimental data obtained with a new method and the reference method (usually an animal test). Similarly, the predictive performances of two (new) methods should be compared taking the BR of their prediction methods into account.

Within a testing strategy using a combination of methods (a so‐called “defined approach,” see Bauch et al., 2012; OECD, 2016), inconclusive results by one (or more) testing methods may still facilitate an unambiguous overall conclusion. Yet it requires defining the prediction model of the testing strategy as a whole. Defined approaches have particularly been used to assess the skin sensitizing properties of chemicals. They combine the data of several nonanimal test methods to conclude a test substance should or should not be regarded as skin sensitizer. The adoption of defined approaches into OECD test guideline is still pending but draft guidelines and supporting documents have become available in September 2019 (OECD, 2019e, 2019f). The work undertaken to draft these documents includes an extensive review of the human and mouse skin sensitization reference data. While for the animal reference data some borderline predictions have been taken into consideration, similar scrutiny has not yet been applied to the *in vitro* data. For instance, of the 21 substances classified false negative against the animal test LLNA, 10 had at least one borderline result *in vitro* (in OECD TG 442C, 442D, or 442E methods) (Kolle, Landsiedel, & Natsch, 2020; OECD, 2019e, 2019f). This underlines the need to implement the quantification of BRs on a broader scale in order to make it meaningful for assessing toxicity testing methods’ precision, and for supporting regulatory decision making on the use or nonuse of hazardous chemicals.

The BR reflects a testing method's uncertainty due to its limited precision. Shading light on precision uncertainty stimulates discussion about what defines a “minimum precision,” and a maximum accuracy of tests (see also the Pham et al., 2019). Our results illustrate that there is no “perfect” or *per se* correct method to determine BRs. The aim of our article is to point toxicologist's attention to test method's uncertainty and to stimulate a discussion on how to account for this uncertainty in a coherent but pragmatic way when selecting appropriate testing methods and assessing the hazard of chemicals.

## 1

The definition of the classification threshold (CT) is a core element of the statistical evaluation of a toxicity testing method. We assume that the CT of a toxicity test is exogenously defined. For hazard classification of chemicals, testing protocols usually define the application of a testing method to a number of chemicals, denoted *i*. For each chemical the test is applied in different concentrations *j*. For determining the BR around the CT the following four statistical methods are suggested and explained below. The notation used for the statistical analysis is presented in Table A1.

**Table A1:** Notation Used for the Statistical Analysis

Notation	Explanation
*T*	Classification threshold in a prediction model of a testing method
*i*	Substance (i=1,…,n)
*j*	Concentration tested per substance *i* $\left(j=1,\text{…},k_{i}\right)$
$r_{i,j}$	Number of replicates per substance *i* and concentration *j*
*l*	Replicate per substance *i* and concentration *j* $\left(l=1,\text{…},r_{ij}\right)$
$y_{i,j,l}$	Test result of substance *i*, concentration *j* and replicate *l*
${\bar{y}}_{i,j}$	Arithmetic mean of test results for substance *i* and concentration *j*

## A1 Pooled Standard Deviation

The pooled standard deviation is a weighted average of the standard deviations of different data sets having different sizes. The pooled standard deviation (*SD_p_*) is across substances *i* and concentrations *j*:

(A.1)
$$SD_{p}=\sqrt{\frac{\sum_{i=1}^{n}\sum_{j=1}^{k_{i}}{\left(r_{i,j}-1\right)}^{*}\sigma_{i,j}^{2}}{\sum_{i=1}^{n}\sum_{j=1}^{k_{i}}\left(r_{i,j}-1\right)}},$$

where $\sigma_{i,j}^{2}$ is the variance of experimental results for substance *i* and concentration *j*:

(A.2)
$$\sigma_{i,j}^{2}=\sqrt{\frac{\sum_{l=1}^{r_{i,j}}{\left(y_{i,j,l}-{\bar{y}}_{i,j}\right)}^{2}}{\left(r_{i,j}-1\right)}}.$$

Based on the *SD_p_*, the BR around the prediction model's classification threshold is given by:

(A.3)
$$BR=\left\{T-SD_{p}\;;T+SD_{p}\right\}.$$

Hence, estimates of the *SD_p_* should be robust.

## A2 Pooled Median Absolute Deviation

In the considered experimental settings one will typically repeat each measurement only a few times, *n* ≤ *5*. Even a small sample may contain aberrant values due to, for example, technical problems or measurement inaccuracies. Thus, the *SD_p_* is highly sensitive to outliers and to nonnormality in the distribution of data. As an alternative to the *SD_p_*, a scale estimator called Mean Average Deviation (MAD) can be applied that is considered more robust. Based on the MAD method, the BR is computed (for each test method), analogously to the *SD_p_*, from the pooled median absolute deviation of a test method's results:

(A.4)
$$MAD_{p}=\frac{\sum_{i=1}^{n}\sum_{j=1}^{k_{i}}{\left(r_{i,j}-1\right)}^{*}MAD}{\sum_{i=1}^{n}\sum_{j=1}^{k_{i}}\left(r_{i,j}-1\right)}.$$

The *MAD* is the median absolute deviation of results for substance *i* and concentration *j*:

(A.5)
$$MAD_{i,j}=\frac{\sum_{l=1}^{r_{i,j}}\left|y_{i,j,l}-{\bar{y}}_{i,j}\right|}{\left(r_{i,j}-1\right)}.$$

The *BR* around the prediction model's CT is, then, determined as follows:

(A.6)
$$BR=\left\{T-MAD_{p}\;;T+MAD_{p}\right\}.$$

## A3 Confidence Interval Approximation of the Pooled Median Absolute Deviation Using the Bootstrap Percentile Method

Suppose 1000 bootstrap replications are collected. After ranking from bottom to the top the 90% confidence interval (CI) is obtained by reading out the sample quantiles corresponding to the given probabilities (0.1; 0.9). In our analysis the upper limit of the 90% confidence interval is used. Hence, the BR is given by

(A.7)
$$BR=\left\{T-CI_{0.9}\;;T+CI_{0.9}\right\}.$$

## A4 90% percentile of all MADs

Instead of averaging experimental estimates the borderline range is computed based on the 90% percentile of the distribution of MADs:

(A.8)
$$BR=\left\{T-MAD_{0.9}\;;T+MAD_{0.9}\right\}$$
